# High Speed Computational Ghost Imaging via Spatial Sweeping

**DOI:** 10.1038/srep45325

**Published:** 2017-03-30

**Authors:** Yuwang Wang, Yang Liu, Jinli Suo, Guohai Situ, Chang Qiao, Qionghai Dai

**Affiliations:** 1 Department of Automation, Tsinghua University, Beijing, 100084, China; 2 Shanghai Institute of Optics and Fine Mechanics, Shanghai, 201800, China

## Abstract

Computational ghost imaging (CGI) achieves single-pixel imaging by using a Spatial Light Modulator (SLM) to generate structured illuminations for spatially resolved information encoding. The imaging speed of CGI is limited by the modulation frequency of available SLMs, and sets back its practical applications. This paper proposes to bypass this limitation by trading off SLM’s redundant spatial resolution for multiplication of the modulation frequency. Specifically, a pair of galvanic mirrors sweeping across the high resolution SLM multiply the modulation frequency within the spatial resolution gap between SLM and the final reconstruction. A proof-of-principle setup with two middle end galvanic mirrors achieves ghost imaging as fast as 42 Hz at 80 × 80-pixel resolution, 5 times faster than state-of-the-arts, and holds potential for one magnitude further multiplication by hardware upgrading. Our approach brings a significant improvement in the imaging speed of ghost imaging and pushes ghost imaging towards practical applications.

Originated in quantum optics^1^,^2^, recently ghost imaging draws wide attentions due to that the single-pixel imaging scheme can acts as a viable alternative when array sensors are unavailable, too expensive or of poor performance. After evolution to classical light then to computational imaging scheme^3^, ghost imaging has been studied widely in the past decade. Due to the intrinsic trading-time-for-spatial-resolvability mechanism of single-pixel imaging, the imaging speed and quality of ghost imaging is closely related to the number of illumination patterns for encoding the scene information, given a specific spatial resolution. To raise the imaging speed and quality, researchers have made great efforts to suppress sensor noise^4^,^5^ or optimize the reconstruction algorithm^6^,^7^,^8^,^9^. Differently, other researchers utilize the information from spatial structure of the target scene to project scene adaptive patterns^10^.

All of the work on computational ghost imaging use a SLM working at tens or hundreds hertz to generate illumination patterns, and the low coding efficiency highly limits the imaging speed and quality and sets back putting ghost imaging into practical use. Recently, researchers attempt to capture dynamic scenes under the single pixel scheme by making full use of the modulation speed of currently available fastest SLM. Matthew *et al*.^11^ achieve 10 Hz 32 × 32-pixel imaging or 2.5 Hz 64 × 64 pixel imaging, with a digital mirror device (DMD) allowing binary patterns to be preloaded and displayed at a maximum rate of 20.7 kHz, which is the toplimit for the modulation speed of current SLM. Suo *et al*.^12^ propose a self synchronized scheme for easy use of such high speed SLM. Later, together with a reconstruction algorithm utilizing spatio-temporal redundancies of nature scenes, Li *et al*.^13^ achieve 16 Hz 64 × 64-pixel computational ghost imaging. In spite of all these great efforts, the illumination patterning is still not fast enough and largely limits the imaging speed of computational ghost imaging.

We notice that the spatial resolution of computational ghost imaging (typically less than 100 × 100 pixels) is much lower than that of SLM (usually higher than 800 × 600 pixels). Unitizing this gap, we propose a sweeping based approach to multiply the illumination modulation speed, by trading off relatively redundant spatial resolution of SLM for a much faster modulation. During the duration of each DMD pattern, we modulate the illumination by driving it sweeping the DMD subregions to produce a series of low resolution patterns.

The scheme is sketched in Fig. 1, two galvanic mirrors rotating around vertical axes are used to build a horizontal scanning device. These two mirrors are kept parallel to each other during rotating, so the beam leaving GM2 keeps parallel to the beam entering GM1 and hits the DMD at the same incident angle, which enables the DMD reflecting back the beam exactly in the opposite direction. In this way, the beam entering and leaving GM1 are along the same line but with opposite directions, and the patterns at different positions of DMD are aligned and in the same propagation direction (see Supplementary Video). In our setup, the illumination pattern is jointly determined by the pattern shown on the DMD and the position of scanning beam on the DMD. The position of the scanning beam on the DMD is determined by the angle of the galvanic mirror, which can be read out from the output voltage from galvanic mirror servo board. We calibrate the mapping between the scanning position and the output voltage of galvanic mirrors by displaying specific patterns and measuring the photodiode output. The detailed description of this calibration method is in Sec. Methods. As for the light source, we use a 532 nm green laser (DJ532-40 from Thorlabs), and the resolution of DMD (Texas Instrument DLP Discovery 4100, 7XGA) is 1024 × 768 pixels with maximum of 20 k hertz projection of binary patterns. GM1 and GM2 are both single axis scanning devices (GVS011 from Thorlabs).

In our setup, the coded patterns are generated by scanning the DMD, and the final modulation frequency of our system is


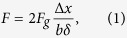


where *F*_*g*_ is the scanning frequency of the two galvanic mirrors GM1 and GM2, and Δ*x* is the scanning range of the beam on the DMD (The detailed description of this variable is in Sec. Methods), *b* is the binning number of DMD mirrors in one direction, and *δ* is the size of each micro-mirror of the DMD. In our setup, *F*_*g*_ is set to be 200 Hz, and the scanning distance Δ*x* is 6.6 mm. The smallest *b* we are using is 2, because imaging at *b* = 1 requires a much more precise mechanical and optical mounting. The size of the DMD mirror is 13.6 *μ*m. Therefore, the binary pattern modulation speed of our system is 97 kHz, which is about 5 times faster than that of the fastest DMD. Please note that our pattern modulation speed is highly limited by the galvanic mirror’s working frequency *F*_*g*_, for some high-end galvanic mirrors with the working frequency achieving 1 kHz e.g. CTI 6200 H, the speed of the same pixel resolution will be as high as 485 kHz. The pixel resolution is mainly determined by the size of the scanning beam, given the specifications of DMD. In our setup, the diameter of the beam is 4mm, and the largest pixel resolution we can achieve is about 200 × 200.

## Results

From Eq. 1, the final illumination patterning speed is independent of the frequency of DMD, but the setting of the DMD’s working frequency is non-trivial. In order to avoid repetitive coding patterns during sweeping of the galvanic mirror pair, the pattern elapse of the DMD should be shorter than the half period of the galvanic mirror. In other words, we can set the DMD at any frame rate between 2*F*_*g*_ and its maximum frequency. However, because the successive scanned sub patterns from a high resolution random pattern is not entirely independent from each other, and this will mathematically degenerate the reconstruction performance. Here we denote the number of scanned consecutive patterns during each DMD period as *k*, and conduct a simulation experiment to test its effects on the final reconstruction and guide the setting of DMD frame rate.

Specifically, we firstly slide a 80 × 80-pixel sub-window over a high resolution random binary pattern to generate *k* small patterns. Then, we use these patterns to code the information of a binary image composed of characters “SINGLE PIXEL”, also with 80 × 80 pixels. The correlated measurements are then generated by their inner products superimposed with white gaussian noise. In this experiment, we vary the parameter *k* from 1 to 100 at interval of 2, and the standard deviation of the noise *σ* increases from 0 to 0.5% at interval of 0.02%. For reconstruction, we utilize compressive sensing (CS) based method^6^ with 35% sample rate, i.e. 2240 patterns. The optimization function is defined as


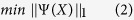



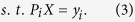


Here *X* denotes the transmission function of the sample, Ψ is the operator transforming the image to a space with sparse representation, and *P*_*i*_ and *y*_*i*_ are the *i*^*th*^ patten and corresponding correlated measurement of PD, respectively. In terms of quantitative evaluation, we adopt RMSE to measure the reconstruction quality. Figure 2(a) visualizes the reconstruction performance at different settings, and some reconstructed images are displayed in Fig. 2(b) with the noise level *σ* = 0.1%. We also simulate the imaging result for a gray-scale scene and show the result on Fig. 2(c) following the same simulation setting with the binary simulation shown in Fig. 2(b). Here *k* = 1 means that all the patterns are independent. It is obvious that the imaging quality turns worse as *k* increases, but even with large noise the result at large *k* still restores a decent image, both visually and quantitatively. In implementation, one should firstly measure the system noise and then choose proper DMD frequency accordingly. In this paper, we set the DMD update frequency as 20*F*_*g*_, which is smaller than the DMD’s maximum frame rate.

Under the above settings, we firstly evaluate the basic imaging performance of the proposed imaging scheme on a static sample, USAF 1951 resolution test chart. The results are shown in Fig. 3, which displays the reconstruction result without scanning (i.e., with both galvanic mirrors hold still) and that with galvanic mirror scanning over the DMD. In implementation, we bin 2 × 2 pixels on the DMD as a super pixel. The pixel resolution of the reconstruction is 80 × 80. For the scanning acquisition, we set DMD working at 4 k Hz and galvanic mirrors working at 200 Hz, and the modulation speed of the illumination is 97 kHz. For the non-scanning acquisition, we set the DMD working at 20 k Hz and the galvanic mirrors working at non-scanning mode. For both experiments, the number of patterns for imaging is 2240. Thus, the acquisition time of scanning version is about 1/5 of that of non-scanning version.From the comparison of the imaging results one can see that our reconstruction is of decent quality, with slightly higher background noise. This is mainly caused by imperfect mechanical modulation and minorly decreased independence among the random patterns. In spite of the performance degradation, experimentally the reconstruction is still of much higher quality than direct reconstruction at five times reduced sampling rate (shown in Fig. 3(b and c)), and this verifies the effectiveness of the proposed sweeping strategy.

To demonstrate the performance of our system working at high frame rate, we conduct single pixel imaging on a dynamic scene. Specifically, we put another DMD on the sample plane, and display video sequences at 42 Hz. Since the DMD projects gray-scale images by temporally multiplexing the binary mirrors which is inconsistent with nature dynamic scenes, we choose to let the DMD work at binary mode. The DMD frames are different from real moving scenes, and the difference is similar to the motion blur of frame-based video recording. For usual scenes not moving quite fast, the video shows no noticeable blur and our demonstration of dynamic imaging is reasonable. The object frames is synchronized by the spike signal in the measurement of the PD, which occurs when DMD refreshes a new frame. For reconstruction, we retrieve each frame using the compressive sensing based algorithm incorporating both spatial and temporal prior constraint, with the optimization function defined as


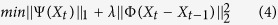



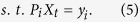


where *X*_*t*_ is the sample at time *t*, Ψ and Φ are transformations applied to the image for sparse representation, and their minimization imposes smoothness constraint on intra and inter frames, respectively. The parameter *λ* is a weighting coefficient balancing two constraints. Empirically, a fast moving scenes favors a small *λ*, and vice versa. One can choose the parameter adaptively as in ref. 13. In this paper, without of loss of generality, we set *λ* = 0.9 empirically according to the dynamics of daily scenes. We show the reconstructed results of two realtime dynamic scenes in Fig. 4. The scenes are projected by another DMD working at 42 frames per second. From the results one can see that our setup can achieve real time single pixel imaging at decent imaging quality. The tiny difference in noise level is due to that the reconstruction algorithm is better at handling the frames with smaller white regions and a simpler structure. This imaging speed is 5 times higher than that of existing counterparts (i.e., DMD’s maximum frequency) and can be accelerated further (please see sec. Discussions), while the previous systems are limited to the patterning frequency of SLM.

In our approach, due to the generating mechanism of illumination patterning, two adjacent patterns are shifted counterparts in the scanning direction (except for the pairs across two DMD periods). For simplicity, we assume the scanning direction is horizontal. Denoting two consecutive patterns as *P*_*i*_ and *P*_*i*−1_, and the pixel indices along horizontal and vertical directions as *u* and *v*, the patterns satisfy *P*_*i*−1_(*u, v*) = *P*_*i*_(*u* − 1, *v*). Let *X* denote the transmission function of the sample, then the correlated measurement for *P*_*i*_ recorded by the photodiode would be *y*_*i*_ = ∑_*u,v*_*P*_*i*_(*u, v)X(u, v*). We do subtraction between *y*_*i*_ and *y*_*i*−1_ and get


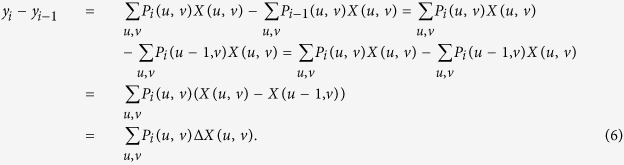


Above derivations tell that, we can reconstruct the edges of *X* from *P*_*i*_, *P*_*i*−1_ and corresponding correlated measurements *y*_*i*_ and *y*_*i*−1_. Utilizing this property, we reconstruct the horizontal edge of two scenes, as displayed in Fig. 5. Here we set the pixel resolution as 80 × 80 pixels, project 2240 patterns and use the same compressive sensing based algorithm as in the simulation experiment. From comparison between (b) (d) and (c) (e), we can see that our approach can easily detect the edges in the target scene. This application holds potential for some computer vision tasks, such as motion detection, tracking, etc. Recall that our detection reduces the number of requisite patterns by 50% compared to the method proposed by Liu *et al*.^14^.

## Discussions

In this paper, we propose to address the speed issue of current computational ghost imaging systems, via bypassing the restrictions from the maximum patterning frequency of SLMs. Specifically, we take advantage of the redundancy of SLM’s spatial resolution and conduct illumination patterning by introducing a pair of galvanic mirrors sweeping periodically over a high resolution SLM. In our proof-of-principle setup, we achieve 5 times faster speed than existing fastest system, and it can be raised further by using higher end elements.

In our system, the speed is largely determined by the operating frequency of galvanic mirror and the size of the DMD micro-mirrors. There are several ways for achieving a much higher efficiency. Firstly, in current setup, we can use a higher end galvanic mirror for further acceleration. For example, using CTI 6200H with 1 kHz working frequency will increase the speed by 5 times, and achieve 210 frames per second at 80 × 80-pixel resolution. Secondly, we can also use an acoustic optical deflectors (AOD) for sweeping, which can produce 20 kHz scanning frequency, and thus 20 times faster illumination patterning, i.e., 9.5 MHz. Note that even with a high end galvanic mirror or AOD, their scanning frequency is sill smaller than half of the DMD’s maximum refreshing rate (i.e., 10 KHz), so the acceleration is feasible in implementation. Thirdly, replacing the DMD with one with smaller mirror size (i.e., *δ*) will bring further acceleration. For example, use the Texas Instrument DLP Discovery 4100,.9XGA (the size of whose micro mirrors is *δ* = 10.8 *μm*) instead of the adopted.7XGA will speed up the imaging by another 20%. In applications, one can choose task specific settings and optical elements.

The pixel resolution is jointly determined by two factors–the size of the SLM entry and that of the scanning beam. Using a DMD with smaller micro mirrors will increase the pixel resolution of the final reconstruction. While under the same scanning rate, we need to trade the final frame rate for higher resolution. Given the DMD specifications (mainly its micro mirror size), the pixel resolution depends on the size of the scanning beam hitting the DMD. The beam size is limited by the size of galvanic mirror. For now, the off-the-shelf galvanic mirrors usually support 3~7 mm beam in diameter, which will provide about 200 × 200-pixel resolution, and this is higher than that of state-of-the-art ghost imaging systems. Usually a larger galvanic mirror supports a lower working operating frequency (i.e., slower modulation), so one need to trade off between the pixel resolution and imaging speed in real applications.

There are also some limitations in our approach. The system needs careful mechanical mount for calibration, but this issue can be addressed effectively by designing a customized programmable mount for the DMD, galvanic mirrors and light source. Another limitation is that currently the scheme works for structuring light using random patterns, and is inapplicable for other patterns with specific structures, e.g. Hadamard and sinusoidal patterns.

## Methods

### Geometry of light path

In our computational ghost imaging system, during each DMD’s pattern duration, the sequential low resolution coded illumination patterns depend on the high resolution pattern on the DMD and the positions (i.e., subregions) where the beam hits the DMD. The hitting position is determined by the rotation angles of the two galvanic mirrors. Here we analyze the geometric relation between the rotating angles of the galvanic mirror pair and the hitting position.

**Figure 1:**
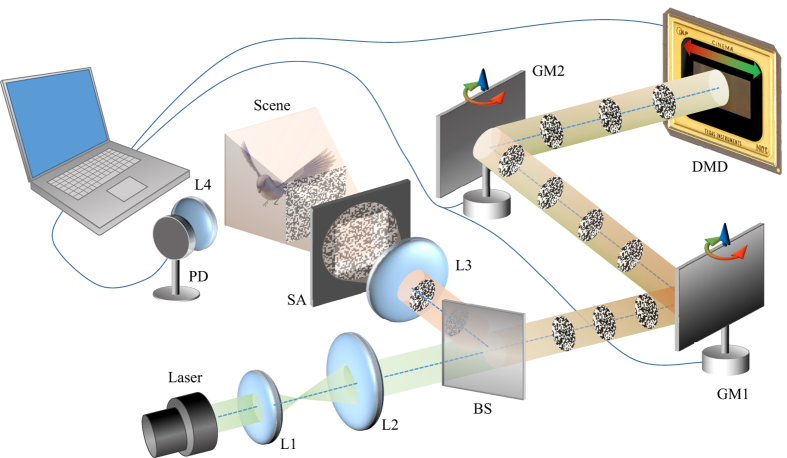
(Better view in color version) Experimental setup for sweeping based high speed ghost imaging system. The laser beam is first collimated and expanded by lens #1 (L1) and lens #2 (L2), then enter the sweeping system which consists of galvanic mirror #1 (GM1) and galvanic mirror #2 (GM2). As the two galvanic mirrors rotate around the vertical axes, the beam sweeping the DMD along the horizontal direction. The beam is coded and reflected back by the digital mirror device (DMD), through GM2 and GM1, then spitted by the beam splitter (BS) and projected onto the scene after being magnified to proper size by lens #3 (L3), and the beam is shaped by a square aperture (SA). The beam entering and leaving the DMD are labeled in light green and light orange colors respectively. Light encoding the scene information is collected by lens (L4) and then recorded by a photodiode (PD). The PD output and GM2’s rotating position are digitized by an analog-to-digital conversion card on the computer for further processing. GM1 and GM2 are set to rotate at the same frequency, amplitude and phase, which ensures the beam between GM2 and DMD being parallel with the beam entering GM1.

**Figure 2:**
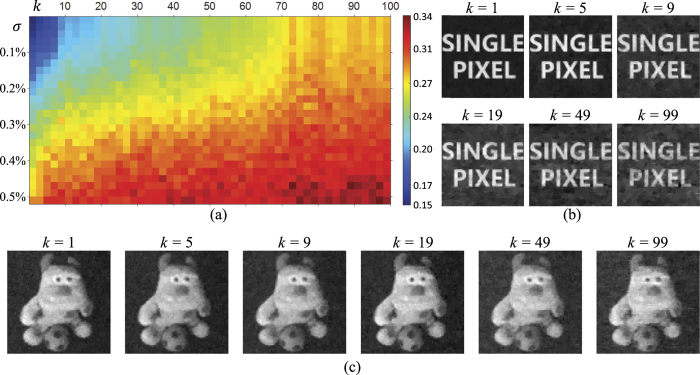
Simulation results on the parameter setting of DMD’s frame rate, specifically the ratio *k* between the frame rates of galvanic mirror and DMD. (**a**) visualizes the performance under varying *k* values at different noise levels with the largest RMSE being 0.44 and smallest RMSE being 0.15. (**b** and **c**) displays some exemplar reconstructed binary and gray-scale images respectively simulated with the same noise level *σ* = 0.1%.

**Figure 3:**
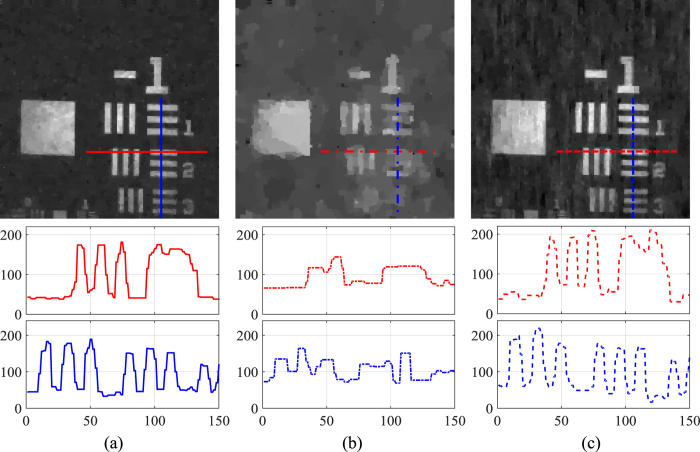
Imaging performance of our setup. The top row of (**a** and **b**) show the reconstruction results of the resolution chart without galvanic mirror scanning using 2240 and 448 patterns respectively. The top row of (**c**) shows the reconstruction result of the resolution chart with galvanic mirror scanning using 2240 patterns. The bottom row of (**a**,**b** and **c**) compare the profile of two local bars of corresponding results plotted in solid, dash-dot and dashed line respectively.

**Figure 4:**
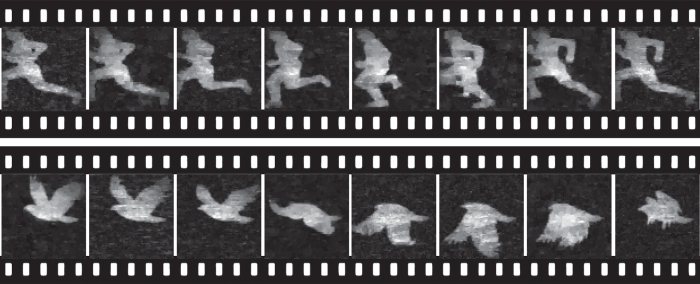
Performance of our setup on two dynamic scenes.

**Figure 5:**
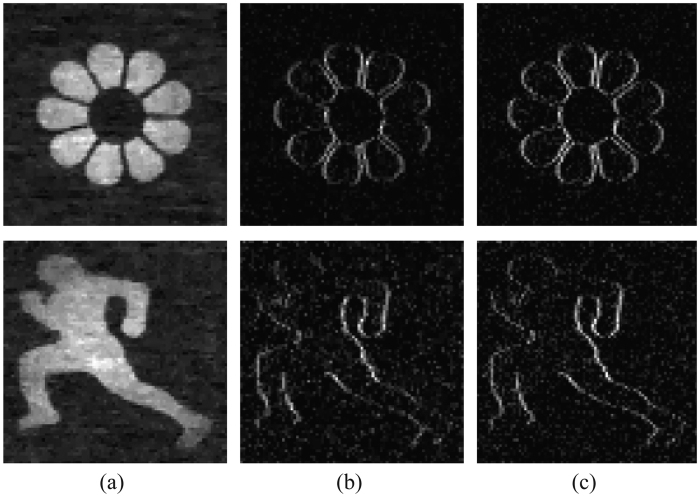
Edge detection result on a scene. (**a**) Imaging result of the sample. (**b**) vs. (**c**) Edge detection results by reconstruction from measurements calculated following Eq. 6 and taking direct horizontal derivative to the image in (**a**).

**Figure 6:**
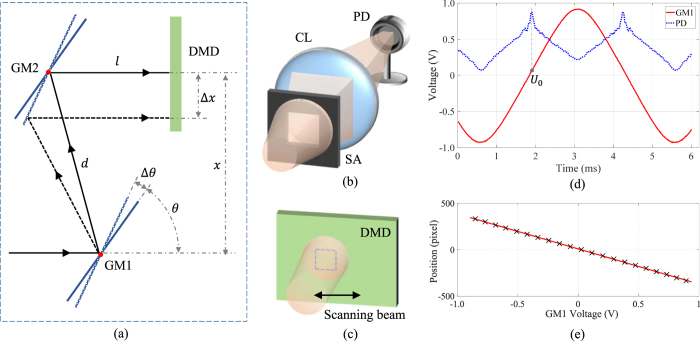
(Better view in color version) (**a**) Geometry of the scanning system with two galvanic mirrors from top view. Galvanic mirror #1 (GM1) and galvanic mirror #2 (GM2) are rotating around the axes denoted by two red points. GM1 and GM2 at rotation angle *θ* and *θ* + Δ*θ* are shown in solid and dashed line respectively. The corresponding beams of *θ* and *θ* + Δ*θ* are also shown in solid and dashed line respectively. Here *d* = 12 cm and *l* = 8 cm. (**b**) The setup for calibration. We shape the beam with a square aperture (SA), and use a converging lens (CL) to collect all the photons to the photodiode (PD). (**c**) A square frame is shown on the DMD which can be covered by the scanning beam at a selected rotating angle. (**d**) The output signal during calibration, red solid line is the output voltage of GM1, and blue dashed curve is the output PD. (**e**) The calibrated mapping between the scanning beam’s hitting position and GM1′s output voltage. Each maker × implies a calibrated mapping pairs between the square border position and related GM voltage output, and the line is linear regression result of those markers.

As illustrated in Fig. 6(a), galvanic mirror #1 (GM1) and galvanic mirror #2 (GM2) rotate around their axes represented as red dots, and the distance between two axes is *d*. We let GM2 always rotated to the same pose with GM1, and thus keep the outgoing beam from GM2 in parallel with the incoming beam to GM1. The distance *d* between the two galvanic mirrors is 12 cm and the distance *l* between DMD and the galvanic mirror 2 is about 8 cm. We use lens #3 (focal length = 15 cm) shown in Fig. 1 to image the DMD onto the object plane. At initial time *t* = 0, the beam is shown by solid black lines, and two galvanic mirrors are shown by solid blue bar. The input voltage of GM1 and GM2 are both initialized to be 0 V, and the position and pose of GM1 and GM2 are adjusted to ensure the beam entering GM1 and GM2 exactly at the position of the rotating axis. Let *θ* denote the rotating angle of GM1, and *x* denote the distance from the beam’s hitting positions at GM1 to that at the DMD, then we can get *x* = *d* *sin*(2*θ*). At time *t* = Δ*t*, the beam is shown by dashed black line, GM1 and GM2 are shown in dotted blue bar. The rotating angle of GM1 and GM2 now is *θ* + Δ*θ*, the distance *x* changes to *x* +Δ*x* correspondingly, with Δ*x* = −*d* *sin*(2Δ*θ*). In our setup, Δ*x* determines the coded pattern of the beam given the image shown on the DMD, and Δ*θ* = *kU*, where *U* is the GM’s input voltage controlling its rotating angle, and *k* is the scaling coefficient between GM’s input voltage and the output rotation angle. Let *p*_0_ be the hitting position (in pixel) at DMD at initial state, and the pixel size of DMD be *δ*_0_, we can obtain the relation between the beam’s pixel position at DMD *p* and the GM’s input voltage *U* as


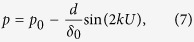


Since the scanning range of GM is small (less than 4 degree), Eq. 7 can be approximated to be


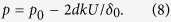


### Calibration

In the calibration step, we build the mapping between the position where the scanning beam is hitting and the related GM’s input voltage *U*. Please note that, since our GM have rotation angle output signal in voltage which is more accurate than input voltage signal, we use this output voltage *U* instead of input voltage. Thus, given an arbitrary *U*, we can retrieve the region on the DMD covered by the scanning beam. The setup for calibration is shown in Fig. 6(b), all the light through the square aperture is collected into the PD directly. Thus at a fixed rotation angle of GM, only the light reflected by a square region on the DMD will reach PD finally. The calibration includes the following two steps: (1) locate the positions of the ends of beam scanning to determine the scanning range; (2) extract mapping relationship between the GM voltage outputs and corresponding position of the square regions within the scanning range.

In the first step, we give a DC voltage to the GM pair, to drive the non-scanning beam hitting two ends of the working region of DMD respectively. Then, at each end position, one-pixel-width horizontal and vertical lines are projected on the DMD to detect the square hitting region of the scanning beam. Since the measurements at the PD will have a step change at the boundary of the hitting region, we can locate the borderlines of the square by thresholding the PD outputs.

In the second step, as illustration in Subsec. Geometry of light path, with a small scanning range of GM, the mapping relationship between the GM voltage outputs and corresponding position of the square regions can be approximated to be linear relationship. We generate a series of square frame patterns of the hitting region size within the range and display them on the DMD sequentially, as Fig. 6(c) shows. During the elapse of each square frame pattern, we drive GM1 and GM2 with sine wave voltages and the measurements of PD changes with the rotation angle of GM1 and GM2. Figure 6(d) plots the PD signals of a frame pattern (in blue dashed curve) and the GM’s output voltage (in solid red line). The peak position implies that the scanning beam hits exactly on the square frame shown on the DMD and reflected beam is through SA. As the dashed lines denotes, the corresponding GM voltage *U*_0_ is recorded as the mapping of the square region. We plot the centroid of each square region and the corresponding GM voltage in Fig. 6(e), from which we can see the linear correlation defined in Eq. 7 is satisfied when the rotation angle is small.

## Additional Information

**How to cite this article**: Wang, Y. *et al*. High Speed Computational Ghost Imaging via Spatial Sweeping. *Sci. Rep.* **7**, 45325; doi: 10.1038/srep45325 (2017).

**Publisher's note:** Springer Nature remains neutral with regard to jurisdictional claims in published maps and institutional affiliations.

## Supplementary Material

Supplementary Video

Supplementary Information
