# Supplementary material for: High Speed Computational Ghost Imaging via Spatial Sweeping
Source: Sci Rep. 2017 Mar 30;7:45325. doi: 10.1038/srep45325 (PMC5372241; doi:10.1038/srep45325)
Supplement: Supplementary Information [file srep45325-s2.pdf]

High Speed Computational Ghost Imaging via Spatial Sweeping

Yuwang Wang[1], Yang Liu[1], Jinli Suo[1], Guohai Situ[2], Chang Qiao[1], and Qionghai Dai[1]

[1] Department of Automation, Tsinghua University, Beijing, 100084, China

[2] Shanghai Institute of Optics and Fine Mechanics, Shanghai, 201800, China

This video illustrates the system setup, the spatial scanning mechanism and modulation procedure of coded illumination. This video will help the reviewers to understand our system better.
